# Cervical Adenitis in Children

**Published:** 1873-02

**Authors:** 


### SELECTIONS.

---

### CERVICAL ADENITIS IN CHILDREN.

BY DR. GUERSANT.

Adenitis of the neck in children is met with so frequently in practice, and so often leaves frightful traces of its invasion, that I deem it expedient to speak of this affection, which is, unhappily, too often seen either neglected or badly treated. Enlargements of the lymphatic glands of the neck, whether in front, at the sides, or on the posterior portion, are sometimes symptomatic of wounds or blows on the scalp or on the face. They are very often the consequence of various affections of the skin of the head or of the face. Occasionally, we can only trace the cause to some disease of the mouth, lips, teeth, or throat. Finally, they may be symptomatic of a more or less lymphatic constitution.

Whatever the causative condition may be, whether general or local, the indication always is at first to attack the cause. If it be a small wound of the head or of the face, we must attend to the wound; if an eczema of the scalp, we must subject it to the proper treatment; if a diseased tooth, and in such a case the glandular enlargement will be seated in the neighborhood of the upper or the lower jaw, the tooth should be extracted. If the cause be a general one, such as a lymphatic constitution, the indication is to prescribe the appropriate remedies. In every case, we must at the same time act locally, either to obtain the resolution of the swelling, or to promote suppuration from it, or to modify the induration, which is a frequent termination of cases of adenitis.

The local symptoms of this affection are sometimes acute, and progress rapidly; at other times, their course is slow or chronic. In all cases, we may recognize three stages: 1. Swelling, redness and increased sensibility over the seat of the ganglions, and, as constitutional symptoms, more or less pronounced fever and *malaise*. 2. Pain, more circumscribed, with augmentation of sensibility, diminution of volume, and often appreciable fluctuation, coming on with more or less rapidity; spontaneous openings, disintegration, fistulæ, irregular scars, slow in their formation; sometimes ulceration of long duration, and more or less disfiguring cicatrization. 3. Diminution of pain, without any notable amount of suppuration; induration, varying in degree, and terminating in a tumor, which gradually disappears by resolution. Finally, the swelling sometimes remains stationary, hard, and free from pain, and may even pass into an enchondromatous condition, but this is only met with in exceptional cases.

1. *To obtain resolution*, we no longer rely, as formerly, on repeated application of leeches; and if, under exceptional circumstances, they seem to be indicated, we should only venture to have recourse to them in cases of adenitis arising from local causes, such as a wound or contusion, and in a person who was not constitutionally lymphatic; otherwise, we should fear that we would obtain only a temporary relief for a few days, while we would retard suppuration, and in all cases weaken the patient.

We depend rather on resolvents, such as pure Neapolitan (mercurial) ointment, combined with extract of belladonna; or else, to replace the mercurial ointment, we would advise the employment of an ointment of calomel. The iodized ointments have not answered so well, but it is always necessary, in using these remedies, to associate them with glycerin, and to observe if they produce redness of the delicate skin of children, when their further use must be suspended and only resumed at a favorable opportunity. Sometimes emollient poultices, made especially of marsh-mallow flour, are of advantage; but if any objection exists to their use, wadding, and even sheep's wool with the grease on, which contains the salts of soda, may be substituted for them. Layers of pure tincture of iodine, applied with a pencil every other day, may hasten the resolution of the swelling. This method is often to be preferred to poultices, which may become sour or grow cold. Such are the means to be adopted to prevent suppuration and induration from occurring.

2. *To hasten Suppuration.*—When the process of suppuration is fairly established, we can no longer rely upon the use of resolvents. Sometimes, but rarely, blisters may succeed in aborting the abscess. We content ourselves then with emollients, usually in the form of general baths and greasy and ripening cataplasms. Finally, when the fluctuation is evident, even before the skin becomes red, we, in common with many of our professional brethren, are favorable to opening the abscess, to prevent spontaneous openings, sometimes multiple, which lead to disintegration of the skin and to frightful scars. We do not absolutely reject the use of the knife, for there are some cases in which we have recourse to it, but we greatly prefer a procedure extolled by MM. Alquié and Bonnafont, namely, the filiform seton, which leaves no traces of the operation. We take for this purpose three or four silk threads, which we pass through the abscess by means of a fine flat needle, in the proper direction, that indicated for the incision, in such a manner that one of the punctures shall be more sloping, and the threads shall be in the direction of the folds of the skin, or following the direction of the muscular fibres, those of the sterno-mastoid, for example. When the little seton, which is tied over as soon as it is passed through, is introduced at the time when fluctuation is evident, the pus will be escaping from the punctures, and its flow may be increased by means of pressure. The application of poultices must be continued, and care taken every day to move the thread. Under this treatment the abscess discharges itself in a few days. The seton should be withdrawn when there is no longer pus or swelling, for, if any tumefaction still exists, its presence hastens the softening of the hard part of the abscess. There remain after this but two punctures, which, at a little later period, leave no traces.

3. *To remove Induration.*—When, in certain cases, the adenitis terminates in induration, we resort to all the resolvents already suggested, iodized ointments, compound plaster of mercury, etc. But if these remedies fail, even when associated with the internal administration of the preparations of iodine, we may employ with advantage the seton, applied in several places, to bring on inflammation and suppuration. We have obtained in this way, by four or five little setons, the suppurative discharge of these chronic cases of adenitis. When enchondroma results, extirpation is the only remedy.

All that we have just said applies to cases of superficial adenitis; but we may add that we have seen quite a number of instances of the same affection deeply seated, even in children at the breast, and other surgeons have mentioned such cases to us. Dr. Fleming and MM. Velpeau and Bouvier have reported similar cases. We have treated several in which the deep ganglions, after being inflamed, commenced to suppurate and to form abscesses along the larynx, the trachea, and occasionally behind the œsophagus and the pharynx. True retro-pharyngeal abscesses may be recognized by a general tumefaction of the neck, which sometimes is more marked on one side than the other, and is occasionally seen on the side of the pharynx. The patient has fever, sometimes delirium, great suffering in moving the neck, difficulty in the use of the voice and in deglutition, and often the inconvenience is so great that asphyxia results. When the patient is old enough to speak, he has a snuffling voice similar to that met with in children with enlarged tonsils. Children have even been brought to me for the purpose of removing their tonsils, who were really suffering from retro-pharyngeal abscesses. In examining the little patient, depress the tongue, and, if abscess is in the median line, a redish, smooth, fluctuating tumor will be detected.

At other times these abscesses, giving rise to a prominence over the lateral portions of the larynx, cause the sterno-mastoid to bulge more or less outwards. In such cases, emollients in the form of general baths, and poultices around the neck, should be applied at the beginning; but as soon as fluctuation is evident, incisions should be made at once, either along the sterno-mastoid, or in the back of the throat over the posterior wall of the pharynx. By such operations the lives of patients are sometimes saved who would otherwise die asphyxiated. Very often these abscesses, being opened, empty themselves completely, and the sufferer is cured without a relapse.

We must not confound these purulent collections which progress quite rapidly with those that are symptomatic of caries of the cervical vertebræ. The latter are developed much more slowly, are always preceded with a different set of symptoms, especially connected with the bony tissues, and are much more serious in their character on account of the destruction of the articulations or of the vertebræ themselves, which are the source of these abscesses.

We need make but brief mention of tuberculous deep-seated lymphatic ganglions, which are developed in the course of the larynx and the trachea. These tumors, scrofulous in their nature, of various consistence, and having a chronic course, are met with in the form of a string of beads on the lateral portions of the respiratory canal, along the great vessels of the neck. They are more or less voluminous and numerous, having the consistence of raw chestnuts or softened horse-chestnuts, and are often confounded with the bronchial glands, which they resemble.

Surgery has sometimes endeavored to remove these degenerated tumors, in order to relieve the patient from the asphyxia which may have resulted. Such operations, which should be rarely attempted, may in the course of their performance expose the sufferer to the greatest dangers, and are often left half-finished. It is much better, then, almost always, in this class of cases, to refrain from the use of the bistoury, and to restrict ourselves to general anti-scrofulous remedies.—*News and Library.*

---

WARTS.—An excellent caustic for cutaneous excrescences is bi-chloro-acetic acid, made by the action of chlorine upon hydrated acetic acid, under the inflnence of solar light. It causes little pain,

